# Differential Contribution of N- and C-Terminal Regions of HIF1α and HIF2α to Their Target Gene Selectivity

**DOI:** 10.3390/ijms21249401

**Published:** 2020-12-10

**Authors:** Antonio Bouthelier, Florinda Meléndez-Rodríguez, Andrés A. Urrutia, Julián Aragonés

**Affiliations:** 1 Research Unit, Hospital Santa Cristina, Research Institute Princesa (IP), Autonomous University of Madrid, 28009 Madrid, Spain; antonio.bouthelier@estudiante.uam.es (A.B.); fcmelrod@gmail.com (F.M.-R.); 2 CIBER de Enfermedades Cardiovasculares, Carlos III Health Institute, 28029 Madrid, Spain

**Keywords:** oxygen, hypoxia-inducible factors, HIF1, HIF2, transcription

## Abstract

Cellular response to hypoxia is controlled by the hypoxia-inducible transcription factors HIF1α and HIF2α. Some genes are preferentially induced by HIF1α or HIF2α, as has been explored in some cell models and for particular sets of genes. Here we have extended this analysis to other HIF-dependent genes using in vitro WT8 renal carcinoma cells and in vivo conditional *Vhl*-deficient mice models. Moreover, we generated chimeric HIF1/2 transcription factors to study the contribution of the HIF1α and HIF2α DNA binding/heterodimerization and transactivation domains to HIF target specificity. We show that the induction of HIF1α-dependent genes in WT8 cells, such as *CAIX* (*CAR9*) and *BNIP3*, requires both halves of HIF, whereas the HIF2α transactivation domain is more relevant for the induction of HIF2 target genes like the amino acid carrier *SLC7A5*. The HIF selectivity for some genes in WT8 cells is conserved in *Vhl*-deficient lung and liver tissue, whereas other genes like *Glut1* (*Slc2a1*) behave distinctly in these tissues. Therefore the relative contribution of the DNA binding/heterodimerization and transactivation domains for HIF target selectivity can be different when comparing HIF1α or HIF2α isoforms, and that HIF target gene specificity is conserved in human and mouse cells for some of the genes analyzed.

## 1. Introduction

In different pathological scenarios such as tumor growth, cardiac ischemia or lung diseases there are insufficiencies in the oxygen supply, a situation that also arises in physiological circumstances like embryonic development. Hypoxia-inducible factors (HIFs) are central to the biological tolerance of hypoxia. HIFs are heterodimeric transcription factors composed of one alpha subunit (HIFα) and one beta subunit (HIFβ), the aryl hydrocarbon receptor nuclear translocator (ARNT) [1,2]. While the HIFβ subunit is stable, the stability of the HIFα subunits is controlled by the prolyl-4-hydroxylase (PHD) domain proteins (PHD1, PHD2 and PHD3), 2-oxoglutarate dependent Fe^2+^-dioxygenases [3,4]. In normoxia, PHDs use oxygen to hydroxylate two conserved proline residues in the HIFα subunits, and these hydroxylated prolyl residues are recognized by the VHL/E3 ubiquitin ligase complex, which targets the HIFα subunits for proteasome degradation [5,6]. Conversely, in hypoxic conditions PHDs do not have enough oxygen to hydroxylate the HIFα subunits, preventing their recognition by VHL/E3 and resulting in their stabilization. Stable HIFα subunits can shuttle to the nucleus, where they can heterodimerize with HIFβ subunits and bind to DNA at the hypoxia response elements (HREs) of target genes, thereby driving a HIF-dependent transcriptional program [7,8,9].

The HIF1α and HIF2α subunits are those that have been studied most intensely, and that have been seen to be involved in numerous cellular responses to hypoxia like angiogenesis, erythropoiesis or metabolic reprogramming [10,11]. Some of the genes induced by HIF can be induced equally by both isoforms, whereas others are preferentially or exclusively controlled by the HIF1α or HIF2α isoform. Indeed, genes encoding glycolytic enzymes are exclusively controlled by HIF1α in different cellular models, which is consistent with the HIF1α isoform participating in the anaerobic metabolic switch executed by hypoxic cells [12,13,14,15]. In sharp contrast, expression of the hypoxia-dependent erythropoietin (*EPO*) gene in kidney tissue is controlled exclusively by the HIF2α isoform [16], reflecting the central role of HIF2α in erythropoiesis. Indeed, EPO production is controlled by HIF2α in scenarios other than the kidney, such as in hepatocytes, astrocytes and pericytes [17,18,19]. Moreover, HIF1α and HIF2α appear to be involved in opposing biological actions, in line with the target genes specifically controlled by each isoform. Thus, HIF1α and HIF2α have contrasting properties in human clear cell renal cell carcinoma (ccRCC), which is characterized by the loss of VHL and the ensuing constitutive stabilization of HIF in normoxic conditions. In this context HIF1α can repress tumor cell proliferation in different biological settings [20,21,22,23], including that of ccRCC, while HIF2α favors the proliferation of *VHL*-deficient RCCs and tumor formation [24,25,26]. These contrasting responses were first related to the distinct effects of HIF1α and HIF2α isoforms on c-Myc activity [24,25]. Moreover other genes have since been shown to be preferentially induced by HIF1α in *VHL*-deficient renal tumor cells, including *carbonic anhydrase IX* (*CAR9*, from here on referred to as *CAIX*) and *BCL2/adenovirus E1B interacting protein 3* (*BNIP3*) [27]. Unlike, some other genes involved in renal cancer proliferation, such as *cyclin D1* (*CCND1*), *transforming growth factor alpha* (*TGFA*) and the amino acid carrier *SLC7A5* are preferentially induced by HIF2α. Thus, these latter genes have been associated with the oncoprotein potential of the HIF2α isoform in *VHL*-deficient RCC [27,28,29].

HIF1α or HIF2α isoforms show a high degree of amino acid similarity in their N-terminal half, the region containing the basic helix-loop-helix (bHLH) domain involved in DNA binding and the Per-Arnt-Sim (PAS) domain that is responsible for heterodimerization with the HIF1β subunit [13,30]. Weaker similarity is found in the C-terminal region of these HIF proteins where their N-terminal transactivation domain (NTAD) and C-terminal transactivation domain (CTAD) transactivation domains are located [13,30]. The specificity of HIF1 for genes like *phosphoglycerate kinase 1* (*PGK-1*) has been attributed to its NTAD region in HEK293 and Hep3B cells [13]. However, both the bHLH-PAS and NTAD/CTAD regions have been shown to be necessary for other HIF1α-dependent genes like *CAIX* in 786-O RCC cells [31,32]. Regarding HIF2α, its NTAD/CTAD region is involved in the HIF2α-dependent induction of the *plasminogen activator inhibitor-1* (*SERPINE1*) and *Cbp/p300-interacting transactivator, with Glu/Asp-rich carboxy-terminal domain 2* (*CITED2*) in some cell lines, such as Hep3B [13], as well as that of *PHD3* in the 786-O cell line [31]. HIF target gene specificity has been largely studied in in vitro cellular models [12,13,27,31,33,34,35]. Moreover, the target specificity of HIF1 and HIF2 in biological settings has been less well explored in vivo [17,36]. In addition, the relative contribution of the bHLH-PAS and NTAD/CTAD halves of HIF1α and HIF2α to target gene selectivity has been studied for particular sets of genes. Here we have extended the analysis to some other HIF1α and HIF2α-dependent genes in an in vitro cell model of human renal cell carcinoma as well as in mice with *Vhl* gene inactivation in which HIF1α and HIF2α isoforms are constitutively activated. Moreover, we have evaluated the relative contribution of the bHLH/PAS and the NTAD/CTAD transactivation halves to confer HIF1α and HIF2α-target selectivity for some genes that have not been included in previous studies. We found that both HIF1α bHLH/PAS and NTAD/CTAD halves can be necessary to induce some HIF1α-dependent genes while the HIF2α NTAD/CTAD half is more relevant to confer HIF2 target selectivity. Finally we found that the target selectivity showed by most of the genes preferentially induced by HIF2α in human renal cell carcinoma is controlled by HIF2α in the liver, the lung and the kidney of *Vhl*-deficient mice, suggesting that HIF target specificity has been conserved in some extent between mouse and human cells.

## 2. Results

### 2.1. Target Gene Selectivity of HIF1α and HIF2α in WT8 Cells

**Figure 1:**
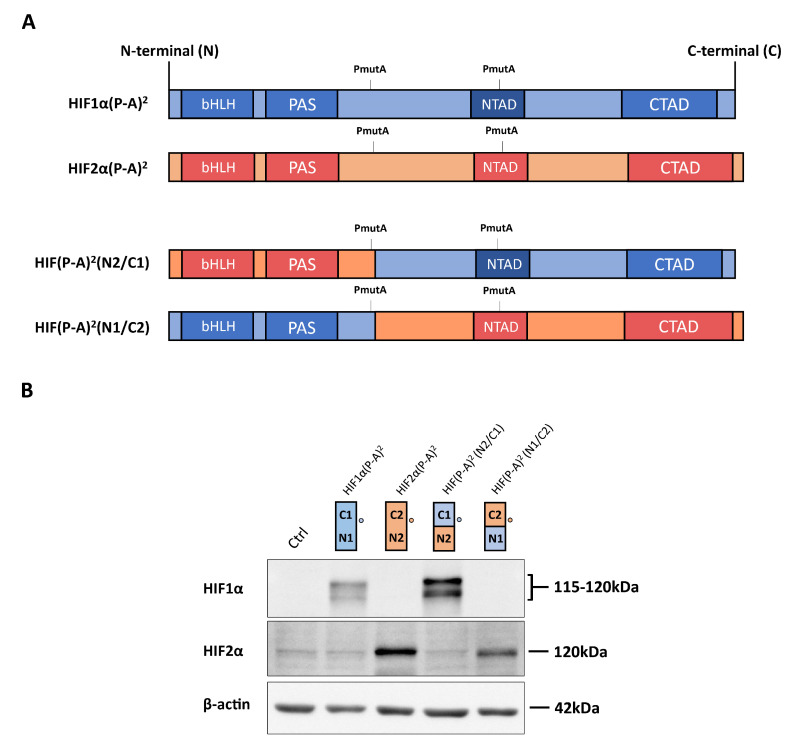
Expression of hypoxia-inducible transcription factor (HIF)1α(P-A)^2^, HIF2α(P-A)^2^ and the HIF(P-A)^2^ (N2/C1), HIF(P-A)^2^ (N1/C2) chimeric versions in WT8 cells. (**A**) Scheme of the HIF1α(P-A)^2^ (in blue) and HIF2α(P-A)^2^ (in red), as well as the HIF1α/HIF2α chimeric constructs. The HIF(P-A)^2^ (N2/C1) construct contains residues 1–414 of HIF2α, including the HIF2α basic helix-loop-helix-Per-Arnt-Sim (bHLH-PAS) domain, and residues 412–826 of HIF1α that includes the HIF1α N-terminal transactivation domain/N-terminal transactivation domain (NTAD/CTAD) transactivation domains. The HIF(P-A)^2^ (N1/C2) construct contains amino acids 1–411 of HIF1α, including the HIF1α bHLH-PAS domain, and amino acids 415–870 of HIF2α that includes the HIF2α NTAD/CTAD transactivation domain; (**B**) representative Western blots probed for the HIF1α, HIF2α and β-actin proteins in control WT8 cells, and those expressing the HIF1α(P-A)^2^, HIF2α(P-A)^2^ and the HIF(P-A)^2^ (N2/C1) or HIF(P-A)^2^ (N1/C2) chimeric constructs. Circle indicates the HIF1α and HIF2α half where the antibodies against HIF1α or anti-HIF2α recognize. Estimated molecular weights of HIF1α and HIF2α (based on molecular weights markers included in the Western blot analysis) are included.

**Figure 2:**
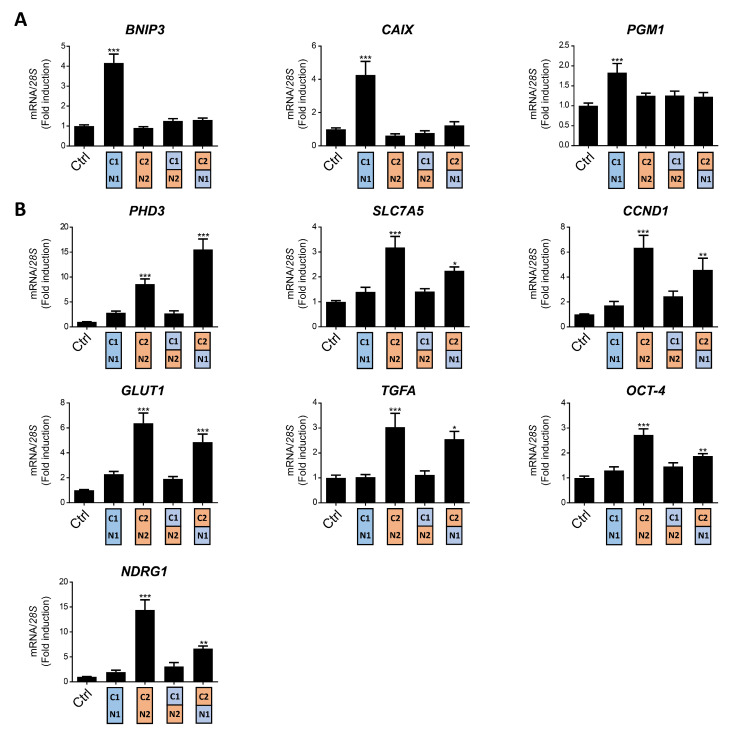
Target gene specificity for HIF1α(P-A)^2^, HIF2α(P-A)^2^ and the HIF(P-A)^2^ (N2/C1), HIF(P-A)^2^ (N1/C2) chimeric versions in WT8 cells. (**A**) Relative *BNIP3*, *PGM1* and *CAIX* gene expression in control WT8 cells and those expressing the HIF1α(P-A)^2^, HIF2α(P-A)^2^ and the HIF(P-A)^2^ (N2/C1) or HIF(P-A)^2^ (N1/C2) chimeric constructs; (**B**) relative *PHD3*, *OCT-4*, *NDRG1*, *TFGA*, *GLUT1*, *CCND1* and *SLC7A5* expression in control WT8 cells (*n* = 6), and those expressing the HIF1α(P-A)^2^ (*n* = 6), HIF2α(P-A)^2^ (*n* = 6) and the HIF(P-A)^2^ (N2/C1) (*n* = 6) or HIF(P-A)^2^ (N1/C2) (*n* = 4) chimeric constructs. Data are shown as mean ± SEM. Statistical analysis was performed using one-way ANOVA followed by Tukey’s post hoc test. * *p* < 0.05, ** *p* < 0.01, and *** *p* < 0.001. Significance with control group is indicated.

The *VHL*-deficient ccRCC cells represent a model in which genes preferentially induced by HIF1α and HIF2α have been identified, and where both HIF isoforms are constitutively active in normoxic conditions. To study the distinct transcriptional responses provoked by HIF1α or HIF2α in renal cell carcinoma (RCC), we used WT8 cells that were generated by restoration of *VHL* expression into the 786-O *VHL* deficient RCC cell line [26]. The specific transcriptional effect of the HIF1α and HIF2α isoforms can be investigated in this cell model by expressing constitutively active HIF1α or HIF2α constructs HIF1α(P-A)^2^ or HIF2α(P-A)^2^, which lack the critical proline residues for VHL recognition (Figure 1A,B). As such, the expression of *CAIX*, *BNIP3* and *phosphoglycerate mutase-1* (*PGM1*) was elevated exclusively in HIF1α(P-A)^2^ WT8 cells but not in HIF2α(P-A)^2^ WT8 cells relative to the control cells (Figure 2A), in line with previous studies showing that these genes are induced by HIF1α in ccRCC cells [27,31,37,38]. By contrast, the expression of other HIF-dependent genes like *PHD3*, *CCND1*, *solute carrier family 2 member 1* (*SLC2A1* or *GLUT-1*), *TGFA*, *POU domain class 5 transcription factor 1* (*POU5F1* or *OCT-4*) and *N-myc downstream regulated gene 1* (*NDRG1*) was preferentially elevated in HIF2α(P-A)^2^ WT8 cells (Figure 2B), again in line with previous studies showing the participation of HIF2α in the gene expression of these genes [27,29,31,39,40].

As described above, there is higher amino acid similarity in the half of the HIFα isoforms involved in DNA binding and heterodimerization, containing the bHLH and PAS domains, than in that which contains their NTAD and CTAD domains (Figure 1A). Therefore, we set out to assess the relative contribution of the bHLH-PAS region as opposed to that of the NTAD/CTAD transactivation region of the HIF1α and HIF2α isoforms to their target gene selectivity in WT8 cells. As such, we generated a HIF(P-A)^2^ (N1/C2) chimera that contained the HIF1α bHLH-PAS N-terminal region (residues 1 to 411 of HIF1α) fused to the HIF2α C-terminal stabilization/transactivation region (residues 415 to 870 of HIF2α) (Figure 1A). Similarly, we also generated the HIF(P-A)^2^ (N2/C1) chimera comprised of the HIF2α bHLH-PAS N-terminal region (residues 1 to 414 of HIF2α) fused to the HIF1α C-terminal stabilization/transactivation region (residues 412 to 826 of HIF1α) (Figure 1A). Like HIF1(P-A)^2^ and HIF2(P-A)^2^, these chimeras also lack the key proline residues for VHL recognition and therefore they are constitutively expressed in WT8 cells under normoxic conditions. Indeed, HIF(P-A)^2^ (N1/C2) and HIF(P-A)^2^ (N2/C1) chimeras were also efficiently expressed in normoxic WT8 cells (Figure 1B). The expression of *CAIX*, *BNIP3* and *PGM1* was not elevated in either HIF(P-A)^2^ (N2/C1) WT8 cells or HIF(P-A)^2^ (N1/C2) WT8 cells (Figure 2A). Hence, these data suggest that the HIF1(P-A)^2^-dependent induction of *CAIX*, *BNIP3* and *PGM1* expression requires the integrity of both the bHLH-PAS and the transactivation NTAD/CTAD HIF1α region (Figure 2A).

In terms of the HIF2-dependent genes, we first found that the HIF (P-A)^2^ (N2/C1) chimera did not induce or produced a modest elevation in the expression of the HIF2-dependent genes analyzed. In contrast *PHD3* expression was induced by the HIF(P-A)^2^ (N1/C2) chimera to a greater extent than by HIF2(P-A)^2^ (Figure 2B). Moreover HIF(P-A)^2^ (N1/C2) also induced *CCND1*, *GLUT1*, *TGFA*, *NDRG1* and *OCT-4* expression but only partially when compared with HIF2(P-A)^2^ especially *OCT-4* and *NDRG1* (Figure 2B). Notably, the HIF(P-A)^2^ (N1/C2) chimeric protein was routinely expressed more weakly than HIF2(P-A)^2^ (Figure 1B), which might also explain the partial induction of *CCND1*, *GLUT-1*, *TGFA*, *NDRG1* and *OCT-4* gene expression by this HIF(P-A)^2^ (N1/C2) chimera (see discussion). These data suggest that the NTAD/CTAD transactivation region of HIF2α plays a role in the gene expression specifically induced by HIF2, especially in the case of *PHD3* for which HIF2α selectivity could be fully attributed to this C-terminal region of HIF2α in WT8 cells. Moreover, these data also suggest that the relative contribution of the NTAD/CTAD transactivation region of HIF2α might be different in each HIF2α-dependent gene.

Collectively these data suggest that selective HIF1-dependent induction of *CAIX*, *BNIP3* and *PGM1* in WT8 cells requires the presence of both the bHLH-PAS and transactivation NTAD/CTAD regions of HIF1α, while the transactivation NTAD/CTAD region of HIF2α seem to be more relevant than the bHLH-PAS region to explain the preferential induction of *PHD3*, *CCND1*, *GLUT-1*, *TGFA*, *OCT-4* and *NDRG1* by HIF2α.

### 2.2. The Role of the HIF2α NTAD/CTAD Transactivation Region in the Expression of the SLC7A5 Amino Acid Carrier

We previously identified the expression of the amino acid carrier SLC7A5 to be preferentially induced by the HIF2α isoform in RCC cells, providing a molecular basis for the pro-proliferative activity of HIF2α in these tumor cells [28]. Moreover, increased SLC7A5 expression has been found in VHL-deficient human ccRCC samples relative to a healthy kidney [28,41,42]. In line with our previous studies, the expression of *SLC7A5* mRNA was preferentially induced in HIF2α(P-A)^2^ WT8 cells when compared to HIF1α(P-A)^2^ and control WT8 cells (Figure 2B). Thus, we wondered about the relative contribution of the HIF2α bHLH/PAS and NTAD/CTAD domains to HIF2α-dependent *SLC7A5* expression. We found that the HIF(P-A)^2^ (N1/C2) chimera partially induced *SLC7A5* gene expression, while HIF(P-A)^2^ (N2/C1) did not show any significant effect on *SLC7A5* expression relative to HIF2(P-A)^2^ in WT8 cells (Figure 2B). Therefore, the NTAD/CTAD HIF2α region appeared to be more relevant to explain the preferential *SLC7A5* expression driven by the HIF2α isoform, which is similar to the data obtained for the *CCND1*, *GLUT-1*, *TGFA*, *OCT-4* and *NDRG1* genes (Figure 2B). However, and as mentioned above, it should be noted that the expression of the HIF(P-A)^2^ (N1/C2) chimera was weaker than that of HIF2(P-A)^2^ (Figure 1B), which might also explain why HIF(P-A)^2^ (N1/C2) might be less potent as HIF2(P-A)^2^ to induce *SLC7A5*.

### 2.3. HIF1α and HIF2α Selectivity in Vhl-Deficient Tissues

**Figure 3:**
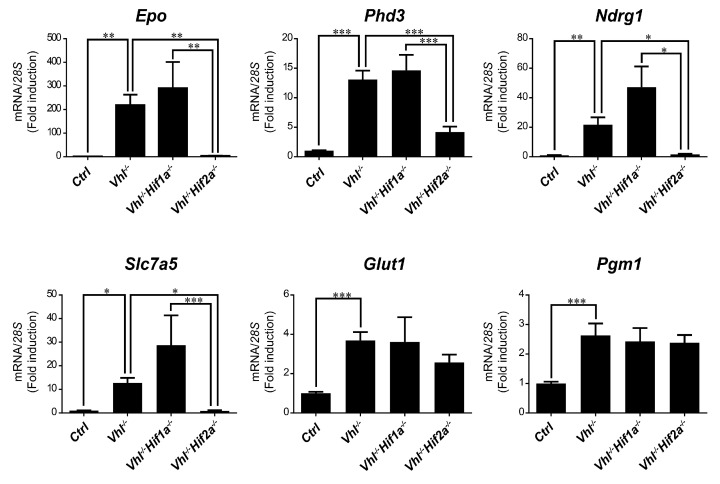
Target gene selectivity for HIF1α or HIF2α in the liver of *Vhl*^−/−^ mice. Relative *Epo*, *Phd3*, *Ndrg1*, *Slc7a5*, *Glut1* and *Pgm1* expression in the liver of *Vhl*^−/−^ mice (*n* = 13–14), *Vhl*^−/−^*Hif1a*^−/−^ mice (*n* = 5–7), *Vhl*^−/−^*Hif2a*^−/−^ mice (*n* = 12) and the corresponding controls (*n* = 15–18). Data are shown as mean ± SEM. Statistical analysis was performed using one-way ANOVA followed by Tukey’s post hoc test. * *p* < 0.05, ** *p* < 0.01, and *** *p* < 0.001.

**Figure 4:**
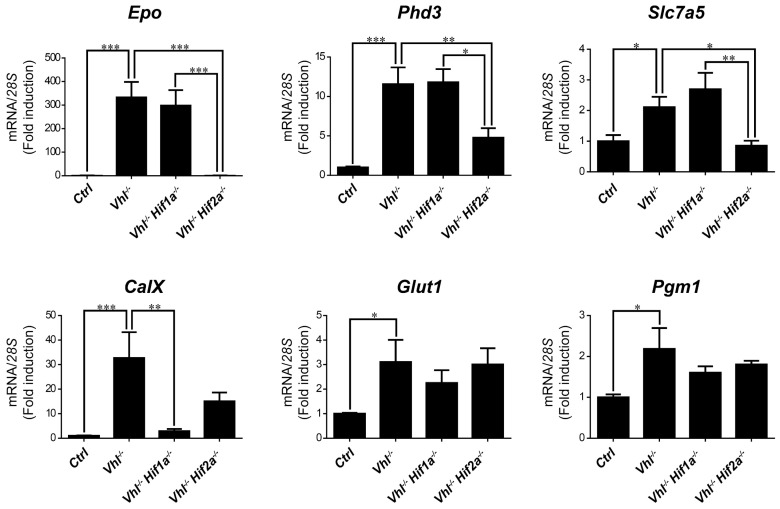
Target gene selectivity for HIF1α or HIF2α in the kidney of *Vhl*^−/−^ mice. Relative *Epo*, *Phd3*, *Slc7a5*, *CaIX, Glut1* and *Pgm1* expression in the kidney of *Vhl*^−/−^ mice (*n* = 6), *Vhl*^−/−^*Hif1a*^−/−^ mice (*n* = 3), *Vhl*^−/−^*Hif2a*^−/−^ mice (*n* = 5) and the corresponding controls (*n* = 8–10). Data are shown as mean ± SEM. Statistical analysis was performed using one-way ANOVA followed by Tukey’s post hoc test. * *p* < 0.05, ** *p* < 0.01, and *** *p* < 0.001.

**Figure 5:**
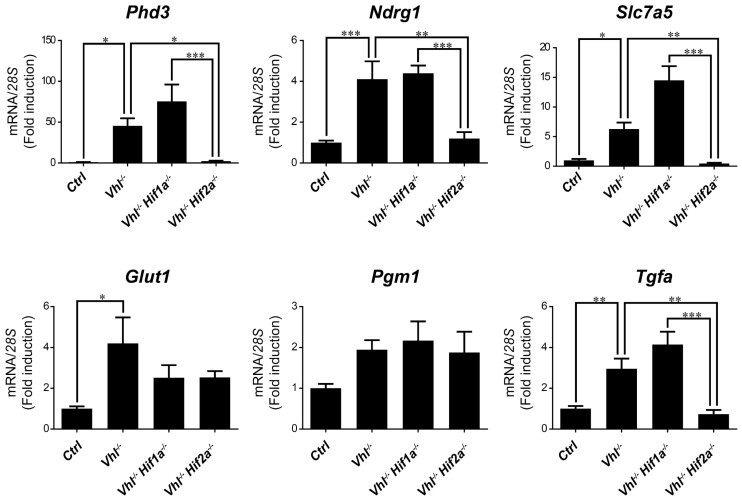
Target gene selectivity for HIF1α or HIF2α in the lung of *Vhl*^−/−^ mice. Relative *Phd3*, *Ndrg1*, *Slc7a5*, *Glut1*, *Pgm1* and *Tfga* expression in the lung of *Vhl*^−/−^ mice (*n* = 4–6), *Vhl*^−/−^*Hif1a*^−/−^ mice (*n* = 5), *Vhl*^−/−^*Hif2a*^−/−^ mice (*n* = 7) and the corresponding controls (*n* = 5–6). Data are shown as mean ± SEM. Statistical analysis was performed using one-way ANOVA followed by Tukey’s post hoc test. * *p* < 0.05, ** *p* < 0.01, and *** *p* < 0.001.

We then asked whether HIF1α and HIF2α selectivity in WT8 RCC cells was also conserved in vivo upon HIF activation in mouse tissues. To this end, we generated adult *UBC*-*Cre-ER^T2^ Vhl^LoxP/LoxP^*-mice (here on are referred to as *Vhl*^−/−^), in which the expression of *Vhl* can be acutely inactivated globally (Supplementary Figure S1), leading to constitutive HIF1α and HIF2α activation [28,43]. In addition, we also generated *Vhl*^−/−^*Hif1a*^−/−^ and *Vhl*^−/−^*Hif2a*^−/−^ mice in which *Vhl* and *Hif1a* or *Hif2a* can be inactivated simultaneously, allowing us to investigate the potential target gene specificity for either HIF isoform (Supplementary Figure S1). As a positive control we first analyzed erythropoietin (*Epo*) mRNA levels in liver and kidney tissue. In line with previous studies *Epo* and mRNA levels are markedly induced in *Vhl*^−/−^ liver (Figure 3) and kidney (Figure 4) through HIF2α isoform [17,44,45,46]. We also found that the expression of *Phd3* was induced consistently in the liver, kidney and lung of both *Vhl*^−/−^ and *Vhl*^−/−^*Hif1a*^−/−^ mice (Figure 3, Figure 4 and Figure 5), indicating that the induction of *Phd3* in vivo was not driven by the HIF1α isoform in these three tissues (Figure 3, Figure 4 and Figure 5). However, elevated expression of *Phd3* was markedly reduced in *Vhl*^−/−^*Hif2a*^−/−^ mice (Figure 3, Figure 4 and Figure 5), indicating that the HIF-dependent expression of *Phd3* in the liver, kidney and lung was driven by the HIF2α isoform, as in WT8 cells. In line with our previous study [28], we also found that *Slc7a5* expression was preferentially induced by the HIF2α isoform in the liver, kidney and lung of *Vhl*^−/−^ mice (Figure 3, Figure 4 and Figure 5), as observed in WT8 cells. However, it should be noted that the increase in *Slc7a5* expression in the kidney tissue takes place to a lesser extent when compared with the liver and lung tissue (Figure 3, Figure 4 and Figure 5). Moreover, *Tgfa* expression was only induced in the lung tissue of *Vhl*^−/−^ mice preferentially by the HIF2α isoform (Figure 5). Furthermore, *Ndrg1* expression was markedly induced in the *Vhl*^−/−^ liver and lung tissue through the HIF2α isoform (Figure 3 and Figure 5). Therefore, *Tgfa* and *Ndrg1* displayed a similar HIF2α specificity in these tissues as in renal cell carcinoma WT8 cells but not induced in the *Vhl*-deficient kidney tissue (see discussion). In contrast, *CaIX* expression was markedly in *Vhl*^−/−^ kidney tissue and reduced to a larger extent in the kidney tissue of *Vhl*^−/−^*Hif1a*^−/−^ than *Vhl*^−/−^*Hif2a*^−/−^ mice (Figure 4). These data suggest that *CaIX* expression is largely controlled by HIF1α in line with data obtained in WT8 cells. Furthermore, while *Glut1* expression was induced in the liver, kidney and lung of *Vhl*^−/−^ mice, its expression was not significantly reduced in both *Vhl*^−/−^*Hif2a*^−/−^ and *Vhl*^−/−^*Hif1a*^−/−^ mice although a trend to be reduced is observed in the liver of *Vhl*^−/−^*Hif2a*^−/−^ mice, the kidney of *Vhl*^−/−^*Hif1a*^−/−^ mice as well as the lung of *Vhl*^−/−^*Hif2a*^−/−^ and *Vhl*^−/−^*Hif1a*^−/−^ mice (Figure 3, Figure 4 and Figure 5). *Pgm1* expression showed a significant induction in *Vhl*^−/−^ liver and kidney tissue (Figure 3 and Figure 4) and a trend in *Vhl*^−/−^ lung tissue (Figure 5). Similar to *Glut1*, the expression of *Pgm1* in the liver and kidney was not affected when compared *Vhl*^−/−^ with *Vhl*^−/−^*Hif2a*^−/−^ and *Vhl*^−/−^*Hif1a*^−/−^ mice (Figure 3 and Figure 4). These data suggest that both HIF1α and HIF2α isoforms might contribute to *Glut1* and *Pgm1* expression in *Vhl*^−/−^ mouse tissues analyzed. Therefore, the HIF selectivity of *Glut1* and *Pgm1* appeared to differ in human WT8 cells to that in mouse tissues of *Vhl*^−/−^ mice.

Together these data indicate a different contribution of the bHLH-PAS and NTAD/CTAD halves of HIF1α and HIF2α isoforms to the specific activity of these factors on their target genes. Moreover, we show that HIF target selectivity is conserved for some—not all—genes when compared to in vivo mouse tissues and a WT8 renal cell carcinoma cell model.

## 3. Discussion

The HIF1α and HIF2α isoforms are central factors in the cellular response to hypoxia. However, HIF1α and HIF2α do not affect all HIF-dependent genes equally. This phenomenon has been investigated in *VHL*-deficient RCC cells characterized by the constitutive activation of HIF1α and HIF2α isoforms, and where each isoform has a distinct biological output. Indeed, some HIF-dependent genes like *CAIX* are preferentially induced by the HIF1α isoform in these RCC cells while other genes like *PHD3* are preferentially induced by the HIF2α isoform [12,13,27,31,33,35].

In this study we show that HIF isoforms specifically target some genes in an RCC model (WT8 cells), as also manifested in other *Vhl*-deficient biological settings in vivo. Indeed, expression of the *Slc7a5* and *Phd3* genes is preferentially induced by the HIF2α isoform in the liver, kidney and lung of *Vhl* deficient mice. In addition, *Tgfa* and *Ndrg1* expression is also preferentially induced by HIF2α but not in all the tissues analyzed. In this line *Tgfa* and *Ndrg1* expression is induced by HIF2α in renal cell carcinoma WT8 cells while is not induced in *Vhl*^−/−^ kidneys. It cannot be ruled out that *Tgfa* and *Ndrg1* expression might be induced in some particular renal cell types in *Vhl*^−/−^ kidneys and therefore could not be detected in this RNA analysis in the whole kidney tissue. Along this line, higher expression of *CaIX*, *Phd3*, *Slc7a5*, *Glut1*, *Pgm1* occurred but not *Ndrg1* in a *Vhl*-deficient renal cell mouse model when compared with non-tumor renal region, which is in line with our data in *Vhl*-deficient kidneys [47]. This study detected an elevated expression of *Tgfa*. This tumor model is a different biological setting than our *Vhl* deficient kidneys. In this line, it might be considered that these tumors are characterized by a specific ccRCC immune microenvironment and that *Vhl* gene inactivation by itself is not enough to initiate the generation of a renal cell carcinoma [48,49,50], which might explain the differences between these two models regarding *Tgfa* expression. Other HIF-dependent genes show different HIF target specificity in mice than in human WT8 cells. For example, *GLUT1* gene expression is preferentially controlled by HIF2α activity in WT8 cells while *PGM1* is preferentially induced by HIF1α isoform. However, our data also show that elevated *Glut1* and *Pgm1* expression in the *Vhl*-deficient mouse tissues analyzed is not significantly reduced upon *Hif1a* or *Hif2a* inactivation, which suggest that both isoforms might be competent to induce *Glut1* and *Pgm1* in both tissues. Along this line, in contrast to renal cell carcinoma cells, *GLUT1* expression has also been shown to be controlled by HIF1α in Hep3B cells [13,32]. Furthermore, *SLC7A5* is preferentially induced by HIF2α in VHL-deficient RCC cells, and in *Vhl*-deficient liver and lung tissue [28,51]. Moreover, HIF2α controls *SLC7A5* expression in other biological settings, such as neuroblastoma cells [52,53]. In addition, *SLC7A5* expression is consistently induced in a panel of breast cancer cell lines subjected to hypoxia [54]. However, glioblastoma cells not only induce *SLC7A5* in response to hypoxia through HIF2α but also, the HIF1α isoform is involved in its expression [55]. The molecular basis of this contrasting HIF selectivity of certain HIF-dependent genes remains unknown. Previous data and those presented here suggest that tissue (or cell) specific factors may influence the participation of HIF1 or HIF2 factors in the regulation of some HIF-dependent genes. In addition the relative abundance of the HIF1α and HIF2α isoforms in each cell type may also contribute in some extent to this HIF target selectivity, particularly if we take into consideration the distinct patterns of HIF1α and HIF2α tissue-specific expression [2,56]. Moreover, differences in HIF selectivity between human and mouse cells cannot be ruled out for some particular HIF-dependent genes.

Previous studies showed that HIF1 target specificity for genes like *CAIX* or *phosphoglycerate kinase 1* (*PGK1*) cannot simply be explained by the preferential binding of HIF1α to the HRE of these two target genes [13,31]. Indeed, HIF2α binds to the HRE of these two genes in hypoxic cells or in VHL-deficient RCC cells [13,31]. Along similar lines, a HIF chimeric protein that contains the bHLH-PAS of HIF1α coupled to the NTAD/CTAD region of HIF2α cannot induce *CAIX* or *PGK1* gene expression in HEK293 cells or RCC cells [13,31,32], highlighting the relevance of the HIF1α NTAD/CTAD region to explain HIF1α target selectivity. Consistent with these data, we also show that a similar HIF (P-A)^2^ (N2/C1) construct cannot induce *CAIX* expression but also, that of other HIF1α target genes like *BNIP3* and *PGM1*. Furthermore, the NTAD region appears to be essential to explain HIF1α target selectivity [13,31,32]. Conversely, a HIF chimeric protein including the bHLH-PAS of HIF2α and the NTAD/CTAD region of HIF1α was sufficient to induce *PGK1* gene transcription in HEK293 cells [13]. These data suggest that the selective *PGK1* expression driven by the HIF1α isoform can largely be explained by the NTAD in HIF1α. However, our data show that the HIF chimera that includes the bHLH-PAS of HIF2α and the NTAD/CTAD region of HIF1α was not capable of inducing *CAIX*, *BNIP3* and *PGM1* expression in WT8 cells. Two independent studies found similar data regarding *CAIX* expression in 786-O RCC and HEK293 cells [31,32]. These data suggest that the bHLH/PAS region may also be relevant to confer HIF1α selectivity to some genes like *CAIX*, *BNIP3* and *PGM1*. In this context, the HIF1α and HIF2α isoform may also show different DNA binding patterns for some genes [57,58] and therefore, some but not all HIF1α selective genes like *BNIP3* or *PGM1* might not bind the HIF2α isoform at their respective promoters. In this line, HIF1α binding to DNA is associated with histone H3K4me3 modifications while HIF2α associates with H3K4me1 [57]. Regarding the additional factors that might help HIF1α to achieve target selectivity, the involvement of the STAT3 transcription factor has been proposed. STAT3 can be recruited specifically to the promoters of HIF1α target genes like *CAIX*, where it contributes to specific HIF1α gene expression [32,59]. Thus, it is possible that STAT3 may also participate in the induction of other HIF1-dependent genes, such as *BNIP3* and *PGM1*.

Previous studies have shown that HIF chimeras that include the HIF1 bHLH-PAS and the HIF2 NTAD/CTAD region induce HIF2α targets genes like *PHD3*, *PAI-1* or adrenomedulin (*ADM*) [13,31,32]. We extended this analysis in WT8 cells to other HIF2 target genes like *OCT-4*, *NDRG1*, *TFGA*, G*LUT1*, *CCND1* and *SLC7A5*. We first found that the expression of these HIF2α target genes is not induced or minimally affected by a chimeric HIF construct that contains the HIF2α bHLH-PAS half and the HIF1α NTAD/CTAD half of the protein. By contrast, a HIF chimera that contains the HIF1α bHLH-PAS half and the HIF2α NTAD/CTAD half is sufficient to induce in a different extent all the HIF2α target genes analyzed, including the amino acid carrier *SLC7A5*, a HIF2α target gene previously identified in different biological settings [28]. However, it should be noted that some of the genes analyzed such as *OCT-4* and *NDRG1* are induced by this chimera to a lesser extent than other HIF2α-dependent genes. These data suggest that the relative contribution of the NTAD/CTAD region of HIF2α to confer target selectivity can be different in each HIF2α-dependent gene. The upstream stimulatory factor 2 (USF2) has been involved in the specific HIF2-dependent expression of *EPO* and *SERPINE1*, involving a physical interaction between the USF2 and the HIF2α NTAD/CTAD region [32,60]. Therefore, it is also possible that USF2 also participates in the HIF2α-dependent expression of *PHD3, OCT-4*, *NDRG1*, *TGFA*, G*LUT1*, *CCND1* and *SLC7A5* in renal cell carcinoma. It should be noted that the HIF chimera that contains the HIF1α bHLH-PAS half and the HIF2α NTAD/CTAD half partially induces the expression of most of the HIF2-dependent genes partially, except *PHD3* that is induced with this chimera at higher levels than HIF2α (P-A)^2^. These data suggest that the HIF2 NTAD/CTAD half might be not sufficient to achieve full HIF2 activity for some HIF2α-dependent genes. However, as indicated above the HIF (P-A)^2^ (N1/C2) construct is routinely expressed more weakly than HIF2α (P-A)^2^, which might also explain the partial induction of HIF2 target genes by the HIF (P-A)^2^ (N1/C2) construct. Nevertheless, we cannot rule out that the induction of a full HIF2 response requires both the HIF2α bHLH-PAS and NTAD/CTAD halves to be present. In this line, it has been proposed that the ETS-1 transcription factor confers HIF2 selectivity by interacting with the bHLH-PAS half of the HIF2α isoform [61]. In addition to ETS-1, Elk-1 is another transcription factor of the ETS family that has been proposed to participate in HIF2 specificity. Again, it is conceivable that the participation of the HIF2 bHLH-PAS region in HIF2 specificity may involve its interaction with ETS-1 and possibly provides a molecular basis of the distinct DNA binding of HIF1α and HIF2α in some HIF-dependent genes referred to above [57].

In general, our data extend the analysis of HIF selectivity to in vivo mouse biological settings where we show that HIF target selectivity can be conserved for some—not all—genes such as *SLC7A5*, *NDRG1* or *TGFA* between human and mouse cells. These data suggest that mechanisms that assure HIF selectivity seem to be conserved during evolution, which might reflect the biological relevance of HIF target specificity. Furthermore, we have shown the involvement of HIF1 bHLH/PAS and NTAD/CTAD regions to confer HIF1α selectivity for the genes analyzed and the major relevance of HIF2α NTAD/CTAD region to understand HIF2 target gene specificity. Further studies will be necessary to understand the conserved molecular basis of HIF target gene selectivity especially using in vivo biological settings.

## 4. Methods

### 4.1. Cell Lines and Cell Culture Conditions

The HEK293T and WT8 cell lines were maintained in Dulbecco’s high glucose modified Eagle’s medium (DMEM: HyClone, GE HealthCare, Chicago, IL, USA) supplemented with 100 units/mL penicillin, 100 μg/mL streptomycin, 20 mM HEPES and 10% fetal bovine serum (U.S.) (FBS: HyClone, GE HealthCare, Chicago, IL, USA). Cells were maintained at 37 °C in an atmosphere of 5% CO_2_/95% air (normoxic conditions).

### 4.2. DNA Plasmid Construction

To generate chimeric HIFα versions, a novel XbaI restriction site was introduced on aa 411 of HA-HIF1α-P402A/P564A and in aa414 of HA-HIF2α-P405A/P531A [62]. For this purpose the N-terminal half of pBabe-puro HA-HIF1α-P402A/P564A (1 to 411) was amplified using a forward primer including an ApaI site (forward, 5′-TTCTCTAgggccc(ApaI)GGCCGGAT-3′) and a reverse primer including an XbaI site (reverse, 5′-TCGTTGCTGCCAAAAtctaga(XbaI)GATATGATTGTGTCTCC-3′). The C-terminal half of pBabe-puro HA-HIF1α-P402A/P564A (412 to 826) was amplified using a forward primer including an XbaI site (forward, 5′-GGAGACACAATCATATCtctaga(XbaI)TTTTGGCAGCAACGA-3′) and a reverse primer including an XbaI site (reverse, 5′-TAACTGACACACATtctaga(XbaI)GGGTCGACCACTGT-3′). The N-terminal half of pBabe-puro HA-HIF2α-P405A/P531A (1 to 414) was amplified using a forward primer including an ApaI site (forward, 5′-TTCTCTAgggccc(ApaI)GGCCGGAT-3′) and a reverse primer including an XbaI site (reverse, 5′-GTTCTGATTCCCGAAAtctaga(XbaI)GAGATGATGGCG-3′). The C-terminal half of pBabe-puro HA-HIF2α-P405A/P531A (415 to 870) was amplified using a forward primer including an XbaI site (forward, 5′-CGCCATCATCTCtctaga(XbaI)TTTCGGGAATCAGAAC-3′) and a reverse primer including an XbaI site (reverse, 5′- TAACTGACACACATtctaga(XbaI)GGGTCGACCACTGT-3′). After confirmation by DNA sequencing each of these four amplicons werecloned in the pCR™2.1-TOPO™ vector (Invitrogen, Carlsbad, CA, USA). Then the N-terminal regions of HA-HIF1α-P402A/P564A and HA-HIF2α-P405A/P531A were excised with ApaI–XbaI to be cloned in pLVX–Puro lentiviral expression vector. Finally, the C-terminal region of HA-HIF1α-P402A/P564A was excised with XbaI–XbaI to be cloned in the pLVX lentiviral expression vector harboring the N-terminal region of HA-HIF2α-P405A/P531A. Similarly, the C-terminal region of HA-HIF2α-P405A/P531A was excised with XbaI–XbaI to be cloned in the pLVX–Puro lentiviral expression vector harboring the N-terminal region of HA-HIF1α-P402A/P564A.

### 4.3. Lentiviral Infection

For lentiviral infection, HEK293T cells were seeded in p100 plates, and transfected using Lipofectamine 2000 (Invitrogen, Carlsbad, CA, USA) with 3.9 μg of pLP1, 2.7 μg of pLP2, 3.3 μg of VSVg and 9.9 μg of each lentiviral vector. Cell culture supernatants were harvested 24 h after transfection, filtered through a 0.45 μm pore filter, and added to WT8 cells along with 8 μg/mL polybrene (final concentration). This step was repeated over the next 2 days and the cells were then selected with 1 mg/mL puromycin to obtain polyclonal resistant cell pools.

### 4.4. Western Blotting and Antibodies

Cells were lysed in Laemmli buffer, and the protein extract was resolved on 10% or 12% SDS-polyacrylamide gels and transferred to 0.45 μm nitrocellulose membranes. The membranes were then blocked and probed with antibodies against: HIF2α (ab199, Abcam); HIF1α (610959, BD Transduction Laboratories, Franklin Lakes, NJ, USA); β-actin (A3854, Sigma, Saint Louis, MO, USA). Antibody binding was detected by enhanced chemiluminiscence (Clarity, BioRad, Hercules, CA, USA; and SuperSignal West Femto Maximum Sensitivity Substrate, Thermo Scientific, Waltham, MA, USA) and visualized on a digital luminescent image analyzer (Image Quant LAS4000 Mini; GE Healthcare, Chicago, IL, USA).

### 4.5. RNA Extraction, RT-PCR Analysis and Primers

Total RNA from the cells was isolated using Ultraspec or TRIsure (BIO-38032, Bioline USA, Inc., Cincinnati, OH, USA). This RNA (1 µg) was then reverse-transcribed using Improm-II reverse transcriptase (Promega, Madison, WI, USA) and polymerase chain reaction (PCR) amplifications were performed using the Power SYBR Green PCR Master Mix kit (Applied Biosystems, Foster City, CA, USA) in a QuantStudio5 (Applied Biosystems, Foster City, CA, USA). Primer sets used are included in Supplementary Table S1. The data were analyzed with QuantStudio5 Design and Analysis Software v1.4 (Applied Biosystems, Foster City, CA, USA).

### 4.6. Mouse Models

C;129S-*Vhl^tm1Jae^*/J (stock no. 4081, Jackson Laboratories, Bar Harbor, ME, USA) were used to generate the *UBC*-*Cre-ER^T2^ Vhl^LoxP/LoxP^* mice. These mice harbor two loxP sites flanking the promoter and exon 1 of the murine *Vhl* locus [63]. The C;129S-*Vhl^tm1Jae^*/J mice were crossed with B6.Cg-*Ndor1^Tg(UBC-cre/ERT2)1Ejb^*/1J or UBC-Cre-ER^T2^ mice (Jackson Laboratories, stock no. 008085) that ubiquitously express a tamoxifen-inducible Cre recombinase (Cre-ER^T2^) [64]. *UBC*-*Cre-ER^T2^ Vhl^LoxP/LoxP^* mice were generated through the appropriate crosses, along with the corresponding control mice. Then *UBC*-*Cre-ER^T2^ Vhl^LoxP/LoxP^Hif1a^LoxP/LoxP^* mice were generated using B6.129-*Hif1a^tm3Rsjo^*/J mice (Jackson Laboratories, stock no. 007561) that harbor two loxP sites flanking exon 2 of the murine *Hif1a* locus [65]. These mice were then crossed with B6.Cg-*Ndor1^Tg(UBC-cre/ERT2)1Ejb^*/1J mice as described above to generate *UBC*-*Cre-ER^T2^ Hif1a^LoxP/LoxP^* mice, which were subsequently crossed with C;129S-*Vhl^tm1Jae^*/J mice to generate *UBC*-*Cre-ER^T2^ Vhl^LoxP/LoxP^Hif1a^LoxP/LoxP^* mice and their corresponding control mice. The *UBC*-*Cre-ER^T2^ Vhl^LoxP/LoxP^Hif2a^LoxP/LoxP^* mice were generated through the appropriate crosses using *Epas1^tm1Mcs^*/J mice (Jackson Laboratories, stock no. 008407) [66].

### 4.7. Ethics Statements

All experimental procedures involving mice were first approved by the research ethics committee at the Autonomous University of Madrid (UAM) (CEIC 55-1002-A049, approval date 9 May 2014 and CEIC 103-1993 -341 approval date 25 November 2019), and they were carried out under the supervision of animal welfare responsible at the UAM in accordance with Spanish RD 53/2013 and European (EU Directive 2010/63/EU) guidelines.

### 4.8. Statistical Analysis

Data were expressed as the mean ± SEM (standard error of the mean), and the differences between groups were analyzed using one-way ANOVA followed by Tukey’s post hoc test. All statistical analyses were performed using GraphPad Prism software (San Diego, CA, USA).

## Supplementary Materials

The following are available online at https://www.mdpi.com/1422-0067/21/24/9401/s1.
