# Supplementary material for: Differential Contribution of N- and C-Terminal Regions of HIF1α and HIF2α to Their Target Gene Selectivity
Source: Int J Mol Sci. 2020 Dec 10;21(24):9401. doi: 10.3390/ijms21249401 (PMC7764359; doi:10.3390/ijms21249401)
Supplement: Supplementary file 1 [file ijms-21-09401-s001.pdf]

Supplementary Materials

Differential Contribution of N- and C-Terminal Regions of HIF1α and HIF2α to Their Target Gene Selectivity

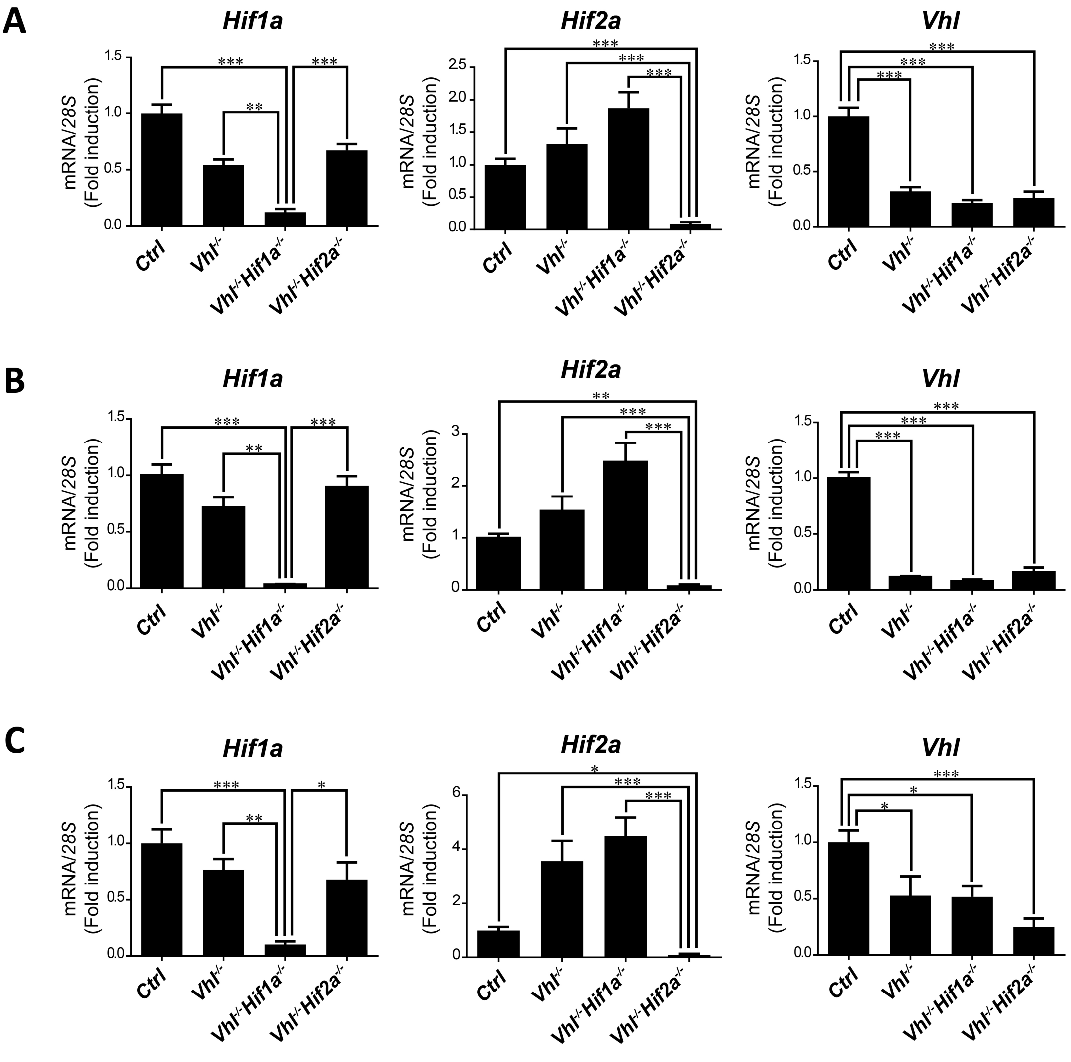

Figure S1. *Vhl*, *Hif1a* and *Hif2a* gene expression in *Vhl*$^{-/-}$, *Vhl*$^{-/-}$*Hif1a*$^{-/-}$ *and Vhl*$^{-/-}$*Hif2a*$^{-/-}$ mice. Relative *Hif1a*, *Hif2a* and *Vhl* expression in the liver (**A**), kidney (**B**) and lung (**C**) of *Vhl*$^{-/-}$ mice (n = 4–14), *Vhl*$^{-/-}$*Hif1a*$^{-/-}$ mice (n = 3–7), *Vhl*$^{-/-}$*Hif2a*$^{-/-}$ mice (n = 5–12) and the corresponding controls (n = 5–18). Data are shown as mean ± SEM. Statistical analysis was performed using one-way ANOVA followed by Tukey's post hoc test. * $p < 0.05$, ** $p < 0.01$, and *** $p < 0.001$.

Table S1. List of human and mouse primers sequences used in the present study.

**HUMAN**		
28S	Forward	5′-GGTAGCCAAATGCCTCGTCAT-3′
	Reverse	5′-GGATAGTAGGTAGGGACAGTGGGAAT-3′
BNIP3	Forward	5′-TCAAGTCGGCCGGAAAATAT-3′
	Reverse	5′-GCGCTTCGGGTGTTTAAAGA-3′
CAIX	Forward	5′-GGGCCCGGAAGAAAACAGT-3′
	Reverse	5′-GACCTGAGTCTCTGAGCCTTCCT-3′
PGM1	Forward	5′-AGCATTCCGTATTTCCAGCAG-3′
	Reverse	5′-GCCAGTTGGGGTCTCATACAAA-3′
PHD3	Forward	5′-GCCGGCTGGGCAAATACTA-3′
	Reverse	5′-CCGGATAGCAAGCCACCAT-3′
SLC7A5	Forward	5′-GGAACATTGTGCTGGCATTATACA-3′
	Reverse	5′-CCTCTGTGACGAAATTCAAGTAATTC-3′
CCND1	Forward	5′-CTGTGCATCTACACCGACAACTC-3′
	Reverse	5′-AGGTTCCACTTGAGCTTGTTCAC-3′
GLUT1	Forward	5′-TCAACCGCAACGAGGAGAA-3′
	Reverse	5′-CTGTCCCGCGCAGCTT-3′
TGFA	Forward	5′-CACTCAGTTCTGCTTCCATGGA-3′
	Reverse	5′-CGTACCCAGAATGGCAG′-3′
OCT-4	Forward	5′-GCTTAGCTTCAAGAACATGTGTA-3′
	Reverse	5′-CTCTCACTCGGTTCTCGAT-3′
NDRG1	Forward	5′-CGGCAACCTGCACCTGTT-3′
	Reverse	5′-TGTGGGTTCCCGGCATT-3′
**MOUSE**		
Vhl	Forward	5′-ATCCCTGAAGAGCCAAAGATGA-3′
	Reverse	5′-TCGACGTTCAGAACTCATCTTTTT-3′
Hif1a	Forward	5′-CACCGATTCGCCATGGA-3′
	Reverse	5′-TCGACGTTCAGAACTCATCTTTTT-3′
Hif2a	Forward	5′-CCTGGCCATCAGCTTCCTT-3′
	Reverse	5′-GGTCGGCCTCAGCTTCAG-3′
Phd3	Forward	5′-TGGACAACCCCAATGGTGAT-3′
	Reverse	5′-GCAGGACCCCTCCATGTAACT-3′
Ndrg1	Forward	5′-TGGAGTCCTTCACCAGTTTGG-3′
	Reverse	5′-CGAAGCGGGTCAGGATGTAG-3′
Slc7a5	Forward	5′-TTCGCCACCTACTTGCTCAA-3′
	Reverse	5′-CCTTTACGCTGTAGCAGTTC-3′
Glut1	Forward	5′-TCGTCGTTGGCATCCTTATTG-3′
	Reverse	5′-GAGCAGCAGAGGCCACAAGT-3′
Pgm1	Forward	5′-CGAGAAGGACGTTGCCAAGA-3′
	Reverse	5′-GGGACACTTTCAGAGCAATGG-3′
Tgfa	Forward	5′-GGCTCTGGAGAACAGCACATC-3′
	Reverse	5′-GGAATCTGGGCACTTGTTGAA-3′
Epo	Forward	5′-CAAAGTCAACTTCTATGCTTGGAAAA-3′
	Reverse	5′-CAGGCCTTGCCAAACTTCTATG-3′
CaIX	Forward	5′-GCGCTAAGCAGCTCCATACTC-3′
	Reverse	5′-CGTGGCTCGGAAGTTCAGTT-3′
